# Urinary Phthalate Metabolite Concentrations and Reproductive Outcomes among Women Undergoing *in Vitro* Fertilization: Results from the EARTH Study

**DOI:** 10.1289/ehp.1509760

**Published:** 2015-11-06

**Authors:** Russ Hauser, Audrey J. Gaskins, Irene Souter, Kristen W. Smith, Laura E. Dodge, Shelley Ehrlich, John D. Meeker, Antonia M. Calafat, Paige L. Williams, for the EARTH Study Team

**Affiliations:** 1 Department of Environmental Health, and; 2 Department of Epidemiology, Harvard T.H. Chan School of Public Health, Boston, Massachusetts, USA; 3 Department of Obstetrics and Gynecology, Harvard Medical School/Massachusetts General Hospital Fertility Center, Boston, Massachusetts, USA; 4 Department of Nutrition, Harvard T.H. Chan School of Public Health, Boston, Massachusetts, USA; 5 Cincinnati Children’s Hospital Medical Center, Cincinnati, Ohio, USA; 6 Department of Environmental Health Sciences, University of Michigan School of Public Health, Ann Arbor, Michigan, USA; 7 National Center for Environmental Health, Centers for Disease Control and Prevention, Atlanta, Georgia, USA; 8 Department of Biostatistics, Harvard T.H. Chan School of Public Health, Boston, Massachusetts, USA

## Abstract

**Background:**

Evidence from both animal and human studies suggests that exposure to phthalates may be associated with adverse female reproductive outcomes.

**Objective:**

We evaluated the associations between urinary concentrations of phthalate metabolites and outcomes of assisted reproductive technologies (ART).

**Methods:**

This analysis included 256 women enrolled in the Environment and Reproductive Health (EARTH) prospective cohort study (2004–2012) who provided one to two urine samples per cycle before oocyte retrieval. We measured 11 urinary phthalate metabolites [mono(2-ethylhexyl) phthalate (MEHP), mono(2-ethyl-5-hydroxyhexyl) phthalate (MEHHP), mono(2-ethyl-5-oxohexyl) phthalate (MEOHP), mono(2-ethyl-5-carboxypentyl) phthalate (MECPP), mono-isobutyl phthalate (MiBP), mono-n-butyl phthalate (MBP), monobenzyl phthalate (MBzP), monoethyl phthalate (MEP), monocarboxyisooctyl phthalate (MCOP), monocarboxyisononyl phthalate (MCNP), and mono(3-carboxypropyl) phthalate (MCPP)]. We used generalized linear mixed models to evaluate the association of urinary phthalate metabolites with in vitro fertilization (IVF) outcomes, accounting for multiple IVF cycles per woman.

**Results:**

In multivariate models, women in the highest as compared with lowest quartile of MEHP, MEHHP, MEOHP, MECPP, ΣDEHP (MEHP + MEHHP + MEOHP + MECPP), and MCNP had lower oocyte yield. Similarly, the number of mature (MII) oocytes retrieved was lower in the highest versus lowest quartile for these same phthalate metabolites. The adjusted differences (95% CI) in proportion of cycles resulting in clinical pregnancy and live birth between women in the fourth versus first quartile of ΣDEHP were –0.19 (–0.29, –0.08) and –0.19 (–0.28, –0.08), respectively, and there was also a lower proportion of cycles resulting in clinical pregnancy and live birth for individual DEHP metabolites.

**Conclusions:**

Urinary concentrations of DEHP metabolites were inversely associated with oocyte yield, clinical pregnancy, and live birth following ART.

**Citation:**

Hauser R, Gaskins AJ, Souter I, Smith KW, Dodge LE, Ehrlich S, Meeker JD, Calafat AM, Williams PL, for the EARTH Study Team. 2016. Urinary phthalate metabolite concentrations and reproductive outcomes among women undergoing in vitro fertilization: results from the EARTH study. Environ Health Perspect 124:831–839; http://dx.doi.org/10.1289/ehp.1509760

## Introduction

Infertility, a disease characterized by the inability to have a child, affects one in six couples and will likely rise as the postponement of childbearing increases in developed regions of the world (Chandra et al. 2005; Evers 2002; Pinnelli and Di Cesare 2005). Even among fertile women, 22% of pregnancies fail before they can be clinically recognized (Wilcox et al. 1988). The associated health care cost of infertility is in the billions of dollars per year and does not include the tremendous physical and psychological burden placed on the couple. This highlights the public health importance of understanding risk factors that may impair the ability to have a child.

It is well appreciated that environmental exposures are potential risk factors for infertility and adverse pregnancy outcomes. Human and experimental animal studies identify several classes of chemicals that adversely impact fertility and pregnancy. Recently, the American College of Obstetricians and Gynecologists (ACOG) and the American Society for Reproductive Medicine (ASRM) issued a Joint Committee Opinion on “Exposure to Toxic Environmental Agents” which emphasized that exposure to environmental chemicals was ubiquitous, and that preconception and prenatal exposure can have a profound effect on reproductive health (ACOG Committee 2013). The Joint Committee Opinion concluded that ACOG and ASRM, along with scientists and other clinical practitioners, noted the need for timely action to identify and reduce exposure to environmental agents while addressing the consequences of exposure.

One class of environmental chemicals for which there is concern related to its risk of adverse reproductive and developmental effects are the ortho-phthalates (herein referred to as phthalates), a family of multifunctional chemicals widely used in personal care and consumer products. Human exposure to phthalates is widespread and occurs through multiple routes, including ingestion, inhalation, dermal contact, and parenteral exposure from medical devices containing phthalates (CDC 2013; Hauser and Calafat 2005). The low-molecular-weight phthalates, such as diethyl phthalate (DEP), di-*n*-butyl phthalate (DnBP), and di-iso-butyl phthalate (DiBP) are principally used in personal care products (e.g., body lotions, cosmetics, shampoos, deodorants) and as solvents and plasticizers for cellulose acetate, varnishes and coatings, including coatings used for time release in some orally administered medications (Hauser and Calafat 2005). High-molecular-weight phthalates [e.g., di(2-ethylhexyl) phthalate (DEHP) and di-isononyl phthalate (DiNP)] are used primarily as plasticizers in the manufacture of flexible vinyl, which, in turn, is used in consumer products, flooring and wall coverings, food contact applications, and medical devices (Hauser and Calafat 2005). Because these phthalate plasticizers are not chemically bound to the polymer chain, they leach, migrate, or off gas, thus leading to human exposure (Koch et al. 2006). Phthalates have a short half-life (i.e., hours) and are rapidly metabolized and excreted in urine and feces (Hauser and Calafat 2005; Silva et al. 2006).

Some phthalates are anti-androgenic and adversely affect the development of the male fetus and male fertility following gestational exposure in the rat to high doses of phthalates (Fisher 2004). However, the impact of phthalates on female reproductive health, fertility, and early pregnancy outcomes is much less studied (Ema et al. 2000; Gray et al. 2006; Lovekamp and Davis 2001; Svechnikova et al. 2007). In the study by Gray et al. (2006), oral administration of DnBP to female Long Evans hooded rats, from weaning through puberty, mating, and gestation, induced mid-pregnancy abortions with up to a 90% reduction in the percentage of females delivering live pups at 1,000 mg/kg/day. The decreased litter size was associated with reduced serum progesterone levels and ovarian progesterone production on gestational day 13. Other studies have shown a reduction in serum levels of progesterone and estradiol in prepubertal female rats dosed with DEHP for 10 days (Svechnikova et al. 2007), and increased post-implantation embryo loss was reported in rats treated with DnBP (Ema et al. 2000).

A limited number of epidemiologic studies have reported associations between measures of exposure to some phthalates with decreased rates of pregnancy and risk of miscarriage, as well as complications such as anemia, toxemia, and preeclampsia in women (Heudorf et al. 2007). A recent human study reported an association between higher urinary concentrations of mono(2-ethylhexyl) phthalate (MEHP), a metabolite of DEHP, and early pregnancy loss (Toft et al. 2012). However, as far as we are aware, no human studies have explored the association of phthalate exposure with very early pregnancy outcomes, such as oocyte fertilization and embryo implantation.

In the present analysis, we assessed data from our ongoing prospective cohort study, the Environment and Reproductive Health (EARTH) study, using assisted reproductive technologies (ART) as a model of human reproduction to explore the relationship between environmental exposures and both male and female reproductive health outcomes.

## Methods

### Participants

Study participants were recruited into the EARTH Study between November 2004 and April 2012 from patients undergoing *in vitro* fertilization (IVF) at the Massachusetts General Hospital (MGH) Fertility Center. All women > 18 years of age and < 46 years (at enrollment) were eligible to participate (approximately 60% of those contacted by the research nurses participated in the study). Women must have contributed their own oocytes and at least one urine sample for the measurement of phthalate metabolites during an IVF cycle to be included in the present analysis. From 429 eligible IVF cycles, we excluded IVF cycles for which women used an egg donor (*n* = 18) or cryo-thaw cycles (*n* = 35), and those missing information on body mass index (BMI) (*n* = 1). This left us with 375 IVF cycles with complete information on exposure, outcome, and all covariates. The EARTH study was approved by the Human Studies Institutional Review Boards of the MGH, Harvard School of Public Health (HSPH), and the Centers for Disease Control and Prevention (CDC). Participants signed an informed consent after the study procedures were explained by a research nurse and all questions were answered.

### Clinical Data

Clinical information at entry into the study and after each IVF cycle was abstracted from the patient’s electronic medical record by the research nurse. At study entry and each IVF cycle, a blood sample was drawn on the third day of the menstrual cycle, and the serum was analyzed for follicle-stimulating hormone (FSH) with an automated electrochemiluminescence immunoassay at the MGH Core Laboratory as previously described (Mok-Lin et al. 2010). At entry into the EARTH Study and subsequent to an infertility evaluation, each patient was given an infertility diagnosis by a physician at the MGH Fertility Center according to the Society for Assisted Reproductive Technology (SART) definitions as previously described (Mok-Lin et al. 2010; SART 2013). At entry, the participant’s date of birth was collected and weight and height were measured by the nurse. BMI was calculated as weight (in kilograms) per height (in meters) squared.

At each IVF cycle, depending on clinical indications and factors such as age and infertility diagnosis, women underwent one of three IVF treatment protocols: *a*) luteal-phase gonadotropin-releasing hormone (GnRH) agonist (low-, regular-, or high-dose leuprolide acetate; Lupron), *b*) follicular-phase GnRH-agonist/Flare stimulation, and *c*) GnRH-antagonist. For each cycle, on the day of ovulation trigger with human chorionic gonadotropin (hCG), serum peak estradiol was measured with an automated electrochemiluminescence immunoassay at the MGH Core Laboratory (Mok-Lin et al. 2010). Couples underwent assisted reproduction with conventional IVF or intra-cytoplasmatic sperm injection (ICSI) (ASRM 2015; Bhattacharya et al. 2001). The ICSI technique was originally developed to treat cases of severe male factor infertility (one or more severely decreased semen parameters) but is now used for certain other indications (Practice Committees of the American Society for Reproductive Medicine and Society for Assisted Reproductive Technology 2012). After egg retrieval, embryologists classified oocytes as germinal vesicle, metaphase I, metaphase II (MII), or degenerated. Embryologists determined fertilization 17–20 hr after insemination. Fertilization was confirmed by the presence of a fertilized oocyte with two pronuclei. Embryos were monitored for cell number and morphological quality [1 (best) to 5 (worst)] on day 2 and 3. For analysis we classified embryos as best quality if they had four cells on day 2, eight cells on day 3, and a morphologic quality score of 1 or 2 on days 2 and 3 (Veeck and Zaninovic 2003). An overall score of 1 or 2 was considered high quality, 3 was considered intermediate quality, and 4 or 5 indicated poor-quality embryos. If a cycle lacked information on day 2 or day 3 embryo quality (e.g., failed fertilization, day 2 transfer), they were classified as having no best-quality embryos. We defined implantation as a serum β-hCG level > 6 mIU/mL typically measured 17 days (range, 15–20 days) after egg retrieval, clinical pregnancy as the presence of an intrauterine pregnancy confirmed by ultrasound at approximately 6 weeks gestation, and live birth as the birth of a neonate on or after 24 weeks gestation. Before the start of the EARTH study, we determined that the infertility medications do not contain phthalates (Kelley et al. 2012), and we tested the IVF equipment and medical supplies for phthalates (data not shown). We did not identify these as potential sources of exposure.

### Urinary Phthalate Metabolite Measurements

Two urine samples were collected during each IVF cycle (between days 3 and 9 of the gonadotropin phase and on the day of the oocyte retrieval). The median time between the two urine samples collected per cycle was 8 days (interquartile range, 6–9), Urine samples were collected between November 2004 and April 2012. Urine was collected in a sterile polypropylene cup. After measuring specific gravity (SG) using a handheld refractometer (National Instrument Company Inc.), the urine was divided into aliquots and frozen at –80°C. Samples were shipped on dry ice overnight to the CDC (Atlanta, GA) for the quantification of concentrations of MEHP, mono(2-ethyl-5-hydroxyhexyl) phthalate (MEHHP), mono-2-ethyl-5-oxohexyl phthalate (MEOHP), mono(2-ethyl-5-carboxypentyl) phthalate (MECPP), mono(3-carboxypropyl) phthalate (MCPP), monocarboxyisooctyl phthalate (MCOP), monocarboxyisononyl phthalate (MCNP), monobenzyl phthalate (MBzP), monoethyl phthalate (MEP), mono-isobutyl phthalate (MiBP), and mono-*n*-butyl phthalate (MBP). The analytical approach, based on solid phase extraction coupled with high performance liquid chromatography-isotope dilution tandem mass spectrometry, followed standard quality assurance/quality control procedures as previously described (Silva et al. 2007). The limits of detection (LOD) were 0.5–1.2 μg/L (MEHP), 0.2–0.7 μg/L (MCOP, MEHHP, and MEOHP), 0.2–0.6 μg/L (MECPP and MCNP), 0.1–0.2 μg/L (MCPP), 0.2–0.3 μg/L (MBzP and MiBP), 0.4–0.8 μg/L (MEP), and 0.4–0.6 μg/L (MBP). We calculated the molar sum of DEHP metabolites (ΣDEHP) by dividing each metabolite concentration by its molecular weight and then summing: {[MEHP × (1/278.34)] + [MEHHP × (1/294.34)] + [MEOHP × (1/292.33)] + [MECPP × (1/308.33)]}. MCPP is a metabolite of di-*n*-octyl phthalate (DnOP) and a nonspecific metabolite of high molecular weight phthalates, MCOP is a metabolite of DiNP, and MCNP is a metabolite of di-isodecyl phthalate (DiDP).

### Statistical Analysis

Demographic characteristics of the study participants and clinical characteristics (cycle- and embryo-level) were reported using mean ± SD or percentages. Cycle-specific urinary concentrations of phthalate metabolites were calculated using the geometric mean (GM) of the two urinary phthalate concentrations from each IVF cycle. Urinary phthalate metabolite concentrations below the LOD were replaced with a value equal to the LOD divided by the square root of 2 (Hornung and Reed 1990). Although there are newer methods to replace values below the LOD (Cole et al. 2009; Nie et al. 2010), our use of quartiles minimized the impact of samples less than LOD; essentially all women who were below the LOD were classified into the first quartile given that the lowest detection limit was 75.5% (for MEHP). For all other metabolites, the lowest quartile included women with concentrations below the LOD and others with low concentrations.

To adjust for urinary dilution, the following formula was used: Pc = P[(1.015 – 1)/SG – 1], where Pc is the SG-corrected phthalate metabolite concentration (micrograms per liter), P is the measured phthalate metabolite concentration (micrograms per liter), and 1.015 is the mean (and median) SG level in the study population (Smith et al. 2012). We used SG-corrected phthalate metabolite concentrations in all analyses.

We fit multivariate generalized linear mixed models with random intercepts to evaluate the association between urinary phthalate metabolites and IVF outcomes. These models allow for the use of multiple outcome observations per individual while accounting for within-person correlations in outcomes. These models are also appropriate and can provide unbiased estimates in the presence of an unbalanced design (e.g., different number of cycles contributed per woman) when imbalance in the number of IVF cycles is not completely random, and the lack of balance can be accurately predicted by all measured covariates in the adjusted model. A linear distribution and identity link function was specified for peak estradiol, a Poisson distribution and log link function were specified for oocyte counts, and a binomial distribution and logit link function were specified for fertilization, embryo quality, and clinical outcomes. Tests for trend were conducted across quartiles using the median urinary phthalate metabolite concentration in each quartile as a continuous variable in the regression models. All results are presented as population marginal means, adjusted for covariates.

Confounding was evaluated using prior knowledge and descriptive statistics from our cohort through the use of directed acyclic graphs. The following covariates were considered for inclusion in the final model: maternal age (continuous), BMI (continuous), smoking status (ever smoked and never smoked), year of treatment (2004–2006, 2007–2009, 2010–2012), day 3 FSH (continuous), treatment protocol type (luteal phase or Flare/GnRH antagonist), and primary infertility diagnosis (female factor, male factor, and unexplained). Variables were included in the final model if they were associated with phthalate exposure in our population, were suspected to be associated with phthalate exposure based on previous research, or were strong predictors of the outcome. To test whether the associations of urinary phthalate metabolite concentrations with IVF outcomes were modified by ICSI, a product of quartiles of phthalate and a binary variable representing the presence or absence of ICSI was entered into the models. Given our limited power, a suggestion of interaction was considered if the *p*-value for this interaction term was < 0.10. As a sensitivity analysis we evaluated the association between urinary phthalate metabolite concentrations and probability of implantation, clinical pregnancy, and live birth per embryo transfer to assess whether any association between urinary phthalates and clinical outcomes remained after excluding early failures. We conducted all statistical analyses using SAS version 9.2 (SAS Institute Inc., Cary, NC) and considered two-sided significance levels < 0.05 as statistically significant.

## Results

**Figure 1:**
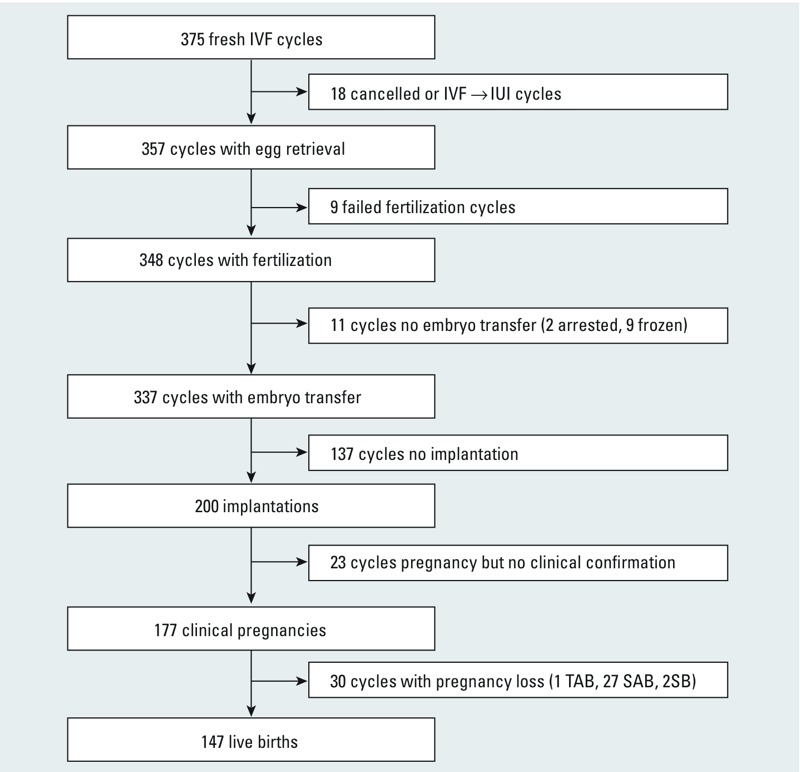
Overview of *in vitro* fertilization (IVF) outcomes of 256 women (375 cycles) in the Environment and Reproductive Health Study enrolled between 2004 and 2012. Abbreviations: IUI, intrauterine insemination; IVF, *in vitro* fertilization; SAB, spontaneous abortion; SB, stillbirth; TAB, therapeutic abortion.

Our analysis included 256 women who were on average 35.3 years of age; 72% had never smoked and 82% were Caucasian (Table 1). The primary SART diagnosis was 37% male factor, followed by 33% unexplained infertility and 30% female factor infertility. The women underwent a total of 375 IVF cycles, with 179 women (69%) contributing 1 cycle, 47 women (18%) contributing 2 cycles, 21 women (8%) contributing 3 cycles, 7 women (3%) contributing 4 cycles, 1 woman (< 1%) contributing 5 cycles, and 1 woman (< 1%) contributing 6 cycles. The luteal-phase treatment protocol was used for 67% of IVF cycles and ICSI (fertilization method) was used for 55% of IVF cycles (Table 2). Among cycles with male factor as the primary infertility diagnosis, 88% used ICSI as compared to 40% and 31% using ICSI with female factor or unexplained infertility diagnoses, respectively. A total of 673 urine samples were collected from the 256 women; 79% of the IVF cycles had 2 urine samples and 21% IVF cycles had only 1 urine sample per cycle. The distribution of each urinary phthalate metabolite in our population is shown in Table 3. An overview of the 375 IVF cycles is shown in Figure 1. In brief, 337 cycles of the initial 375 cycles underwent embryo transfer (90%). Of the cycles that underwent embryo transfer, the percent resulting in implantation, clinical pregnancy, and live birth were 59%, 53%, and 44%, respectively.

**Table 1:** Demographic characteristics and primary SART diagnosis among 256 women in the Environment and Reproductive Health Study enrolled between 2004 and 2012.

Characteristic	*n* (%)
Age at study entry (years)
Mean ± SD	35.3 ± 3.93
Range	21–43
Age ≥ 37	95 (37%)
BMI (kg/m^2^)
Mean ± SD	24.1 ± 4.34
Range	16.1–42.4
Underweight or normal (< 25)	180 (70%)
Overweight or obese (≥ 25)	76 (30%)
Smoking
Never smoked	185 (72%)
Ever smoked
Current smoker	6 (2%)
Former smoker	65 (26%)
Race
Caucasian	211 (82%)
Black/African American	6 (2%)
Asian	21 (8%)
Other	18 (7%)
Primary SART diagnosis at study entry
Female factor	77 (30%)
Diminished ovarian reserve	18 (7%)
Ovulation disorders	22 (9%)
Endometriosis	17 (7%)
Uterine disorders	2 (1%)
Tubal factor	18 (7%)
Male infertility	95 (37%)
Unexplained	84 (33%)
Year at study entry
2004–2006	49 (19%)
2007–2009	115 (45%)
2010–2012	92 (36%)
Abbreviations: BMI, body mass index; SART, Society for Assisted Reproductive Technology (SART 2013).	

**Table 2:** Cycle-specific clinical characteristics from 375 IVF cycles among 256 women in the Environment and Reproductive Health Study enrolled between 2004 and 2012.

Cycle-specific characteristics (*n *= 375 IVF cycles)	*n* (%) or mean ± SD (range)
Treatment protocol
Luteal phase	251 (67)
Flare	77 (20)
Antagonist	47 (13)
Number of embryos transferred
No embryos transferred	38 (10)
1 embryo	46 (12)
2 embryos	206 (55)
≥ 3 embryos	85 (23)
Embryo transfer day
No embryos transferred	38 (10)
Day 2	18 (5)
Day 3	215 (57)
Day 5	104 (28)
Fertilization protocol (*n *= 357)
ICSI	196 (55)
Traditional IVF	161 (45)
Controlled ovarian hyperstimulation outcomes (*n *= 357 IVF cycles^*a*^)
Day 3 FSH (IU/L)	7.15 ± 2.17 (0.2–15.2)
Peak estradiol (pg/mL)	2,071 ± 853 (551–5,263)
Total number of oocytes retrieved	10.91 ± 5.41 (1–32)
Mature (MII) oocytes retrieved	9.20 ± 4.65 (0–27)
Maturation rate (MII oocytes/total oocytes retrieved)	0.86 ± 0.16 (0–1)
Normal (2PN) fertilized oocytes	6.37 ± 3.66 (0–21)
Fertilization rate (2PN/MII oocytes)	0.69 ± 0.24 (0–1)
Total embryos	5.99 ± 3.75 (0–21)
Best embryos	1.48 ± 1.90 (0–13)
Pregnancy outcome (*n *= 375 IVF cycles)
No oocytes retrieved	18 (4.8)
Fertilization failure	9 (2.4)
Arrested embryo development or all embryos frozen	11 (2.9)
Implantation failure^*b*^	135 (36.0)
Chemical pregnancy^*c*^	21 (5.6)
Ectopic pregnancy	4 (1.1)
Spontaneous abortion	27 (7.2)
Therapeutic abortion	1 (0.3)
Stillbirth	2 (0.5)
Live birth	147 (39.2)
Abbreviations: FSH, follicle-stimulating hormone; ICSI, intra-cytoplasmic sperm injection; IVF, *in vitro* fertilization; MII, mature oocytes; PN, pronuclei. ^***a***^Fresh IVF cycles with successful egg retrieval (missing cycles include cycles failing before egg retrieval). ^***b***^Implantation failure was defined as a negative pregnancy test (βhCG < 6 mIU/mL) 17 days following embryo transfer or insemination. ^***c***^Chemical pregnancy was defined as implantation with no subsequent clinical pregnancy.	

**Table 3:** Distribution of urinary phthalate metabolite concentrations (metabolite or molar sum) measured among 256 women in the Environment and Reproductive Health Study enrolled between 2004 and 2012.

Phthalate	Units	*n*^*b*^	LOD	% Detect^*c*^	SG-adjusted		Unadjusted^*a*^	
					Median (IQR)	Maximum	Median (IQR)	Maximum
∑DEHP metabolites	μmol/L	375	—	—	0.19 (0.10, 0.42)	5.60	0.20 (0.09, 0.44)	7.52
MEHP	μg/L	375	0.5–1.2	77.1	2.88 (1.37, 6.87)	99.3	2.72 (1.30, 6.91)	130
MEHHP	μg/L	375	0.2–0.7	99.7	15.7 (7.75, 35.0)	561	16.3 (7.07, 38.2)	582
MEOHP	μg/L	375	0.2–0.7	99.6	10.5 (5.48, 25.4)	306	11.3 (4.92, 25.7)	387
MECPP	μg/L	375	0.2–0.6	96.7	26.3 (14.6, 57.2)	761	29.2 (12.9, 62.9)	1,190
MEP	μg/L	375	0.4–0.8	100	49.3 (21.5, 129)	3,481	52.2 (20.8, 133)	2,537
MBP	μg/L	375	0.4–0.6	97.0	12.9 (7.32, 20.8)	435	12.6 (6.40, 26.3)	250
MCPP	μg/L	375	0.1–0.2	96.7	3.22 (1.80, 6.64)	202	3.63 (1.77, 7.76)	179
MiBP	μg/L	375	0.2–0.3	96.7	6.66 (3.50, 12.0)	49.7	7.03 (3.14, 14.6)	76.3
MBzP	μg/L	375	0.2–0.3	95.5	3.44 (1.75, 7.35)	121	3.99 (1.57, 7.60)	189
MCOP	μg/L	337	0.2–0.7	98.6	22.8 (9.10, 57.7)	1,350	22.7 (8.00, 66.2)	2,250
MCNP	μg/L	337	0.2–0.6	96.6	4.58 (2.51, 7.97)	281	4.75 (2.41, 9.18)	281
Abbreviations: IQR, interquartile range; LOD, limit of detection; *n*, number of IVF cycles; SG, specific gravity. ^***a***^Unadjusted concentrations presented to facilitate comparison with other studies. ^***b***^Geometric mean of up to 2 urinary phthalate concentrations from each IVF cycle was used. ^***c***^Percent of phthalate metabolite concentrations above the detection limits (*n *= 673 total urine samples except for MCOP and MCNP, which have 646 urine samples); total samples < LOD by analyte: MEHP = 154, MEHHP = 2; MEOHP = 3, MECPP = 22, MEP = 0, MBP = 20, MCPP = 22, MiBP = 22, MBzP = 30, MCOP = 9, MCNP = 22.								

**Figure 2:**
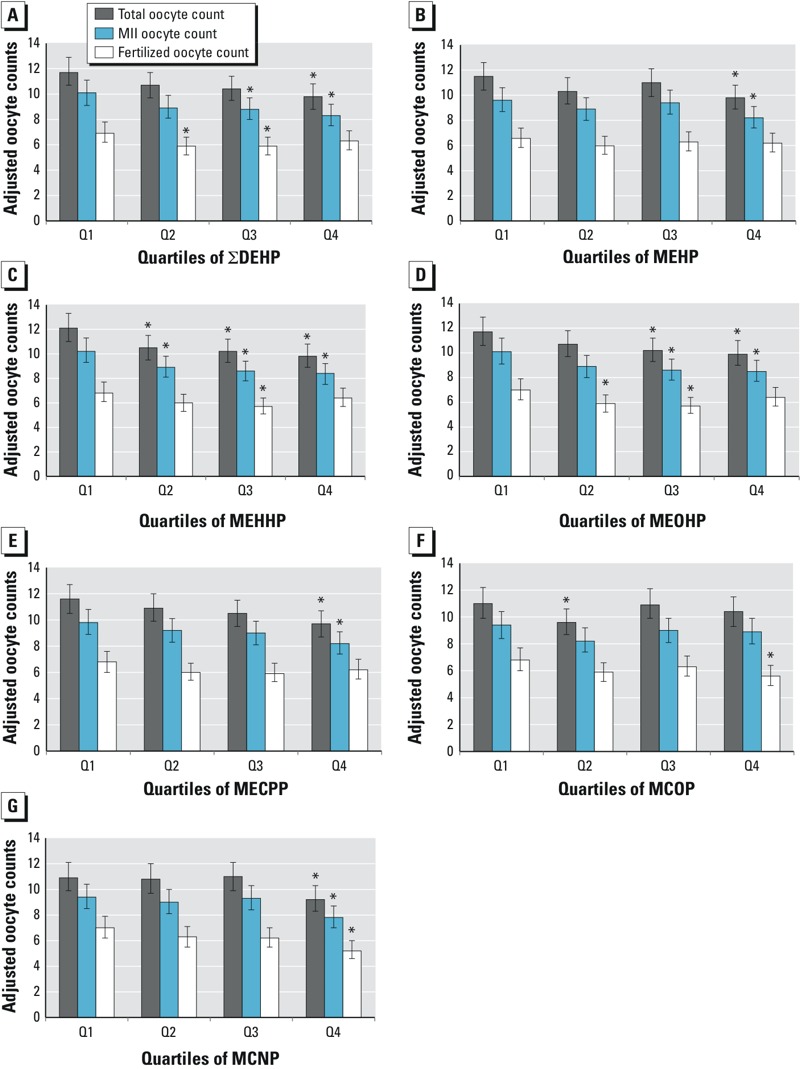
Adjusted mean (95% CI) total, mature (MII), and fertilized oocyte count by quartile of urinary phthalate metabolites and ∑DEHP concentrations among 247 women undergoing 357 IVF cycles with successful egg retrieval: (*A*) ∑DEHP, (*B*) MEHP, (*C*) MEHHP, (*D*) MEOHP, (*E*) MECPP, (*F*) MCOP, (*G*) MCNP. Adjusted models control for maternal age (continuous), body mass index (continuous), smoking status (never, ever), and primary SART infertility diagnosis at study entry (female, male, unexplained). *p-value for comparison against Q1 < 0.05.

In multivariable models adjusted for age, BMI, smoking status, and infertility diagnosis, only MCNP was associated with peak estradiol (*p*-trend = 0.009). The adjusted mean peak estradiol was 2,225 μg/L [95% confidence interval (CI): 2,036, 2,414] in quartile (Q) 1 compared to 1,902 μg/L (95% CI:1,710, 2,094) in quartile 4 (*p*-value for Q4 vs. Q1 = 0.02) (results not shown). In adjusted multivariable models, there were statistically significant associations of ΣDEHP, MEHP, MEHHP, MEOHP, MECPP, and MCNP with reduced total oocyte yield for the 4th compared with the 1st quartile (Figure 2). Consistent with the decrease in oocyte yield, there were decreased numbers of MII oocytes retrieved in quartile 4 compared with quartile 1 for ΣDEHP, MEHP, MEHHP, MEOHP, MECPP, and MCNP (Figure 2). The total number of fertilized oocytes was significantly lower in quartile 4 compared with quartile 1 for MCOP and MCNP, but not the other phthalate metabolites (Figure 2). There was no interaction between use of ICSI and the association of phthalates with the number of fertilized oocytes (data not shown, *p*-value for interaction > 0.10). In multivariable models there were no statistically significant associations of any phthalate metabolite with percent of cycles with one or more high-quality embryos (*p* for trend > 0.05 for all metabolites, and no point estimate comparisons were significant) (data not shown).

After controlling for age, BMI, smoking status, and primary infertility diagnosis, increased quartiles of ΣDEHP were associated with a reduced probability of clinical pregnancy and live birth such that there appeared to be monotonic downward trend (*p* for trend = 0.04 and 0.01, respectively) (Table 4). The adjusted difference (95% CI) in proportion of cycles resulting in clinical pregnancy between women in the highest DEHP quartile (4th) versus lowest quartile (1st) was –0.19 (–0.29, –0.08), representing a decrease in the probability of clinical pregnancy from 0.57 to 0.38 (*p* for trend = 0.04). Similarly, women in this highest ΣDEHP quartile versus the lowest had a mean decrease in proportion of cycles resulting in live birth of 0.19 (–0.28, –0.08), representing a decrease from 0.47 to 0.28, respectively (*p* for trend = 0.01). There was a similar monotonic downward trend in proportion of cycles resulting in clinical pregnancy and live birth across increasing quartiles of individual DEHP metabolites, MEHP, MEHHP, MEOHP, and MECPP (Table 4). The monotonic downward trends between DEHP metabolites and implantation, clinical pregnancy and live birth were similar when analyses were restricted to cycles with embryo transfer (*n* = 337 cycles) (data not shown). Although concentrations of the other phthalate metabolites measured (MEP, MBP, MIBP, MBzP, MCPP, MCOP, and MCNP) were not significantly related to clinical outcomes following IVF (Table 4), 17 of the 18 adjusted models for probability of implantation, clinical pregnancy, and live birth showed reduced proportions for quartile 4 compared with quartile 1. Finally, across all the urinary phthalate metabolites, there were no interactions between the use of ICSI and associations of phthalates with live birth (data not shown).

**Table 4:** Unadjusted and adjusted mean proportion (95% CI) of cycles resulting in implantation, clinical pregnancy, and live birth by quartile of urinary DEHP metabolites and ∑DEHP concentrations among 256 women undergoing 375 IVF cycles.

Phthalate measure quartiles	Implantation		Clinical pregnancy		Live birth	
	Unadjusted	Adjusted	Unadjusted	Adjusted	Unadjusted	Adjusted
∑DEHP metabolites (μmol/L)
Q1 (≤ 0.10)	0.63 (0.52, 0.72)	0.62 (0.50, 0.72)	0.59 (0.48, 0.69)	0.57 (0.45, 0.67)	0.50 (0.39, 0.60)	0.47 (0.36, 0.58)
Q2 (0.11–0.19)	0.54 (0.44, 0.65)	0.55 (0.44, 0.65)	0.46 (0.36, 0.57)	0.46 (0.36, 0.57)	0.44 (0.33, 0.54)	0.43 (0.32, 0.54)
Q3 (0.20–0.42)	0.51 (0.41, 0.62)	0.52 (0.41, 0.63)	0.48 (0.38, 0.59)	0.49 (0.38, 0.59)	0.39 (0.29, 0.50)	0.39 (0.29, 0.50)
Q4 (0.43–5.60)	0.48 (0.38, 0.59)*	0.49 (0.39, 0.60)	0.38 (0.28, 0.48)*	0.38 (0.28, 0.49)*	0.28 (0.20, 0.39)*	0.28 (0.19, 0.39)*
*p*-Trend^*a*^	0.09	0.18	0.02	0.04	0.005	0.01
MEHP (μg/L)
Q1 (≤ 1.36)	0.61 (0.50, 0.70)	0.60 (0.49, 0.70)	0.55 (0.45, 0.65)	0.54 (0.43, 0.64)	0.47 (0.37, 0.58)	0.45 (0.35, 0.56)
Q2 (1.37–2.88)	0.57 (0.46, 0.66)	0.56 (0.45, 0.66)	0.53 (0.42, 0.63)	0.52 (0.41, 0.62)	0.46 (0.35, 0.56)	0.44 (0.34, 0.55)
Q3 (2.89–6.80)	0.47 (0.37, 0.57)	0.48 (0.37, 0.58)	0.43 (0.33, 0.53)	0.43 (0.33, 0.54)	0.37 (0.27, 0.48)	0.36 (0.27, 0.47)
Q4 (6.87–99.3)	0.52 (0.42, 0.63)	0.54 (0.43, 0.65)	0.40 (0.30, 0.50)*	0.41 (0.31, 0.52)	0.30 (0.22, 0.41)*	0.30 (0.21, 0.41)
*p*-Trend	0.43	0.68	0.04	0.09	0.02	0.04
MEHHP (μg/L)
Q1 (≤ 7.74)	0.61 (0.50, 0.70)	0.58 (0.47, 0.69)	0.58 (0.47, 0.68)	0.55 (0.44, 0.66)	0.58 (0.47, 0.68)	0.45 (0.34, 0.56)
Q2 (7.75–15.4)	0.57 (0.46, 0.66)	0.57 (0.47, 0.67)	0.49 (0.39, 0.60)	0.50 (0.39, 0.60)	0.49 (0.39, 0.60)	0.46 (0.36, 0.57)
Q3 (15.5–34.5)	0.51 (0.41, 0.62)	0.52 (0.42, 0.63)	0.46 (0.36, 0.57)	0.47 (0.36, 0.58)	0.46 (0.36, 0.57)	0.35 (0.25, 0.46)
Q4 (34.6–561.3)	0.48 (0.38, 0.58)	0.49 (0.39, 0.60)	0.38 (0.28, 0.48)*	0.38 (0.28, 0.49)*	0.38 (0.28, 0.48)*	0.30 (0.21, 0.41)
*p*-Trend	0.11	0.22	0.01	0.04	0.01	0.03
MEOHP (μg/L)
Q1 (≤ 5.44)	0.60 (0.49, 0.69)	0.58 (0.47, 0.68)	0.58 (0.47, 0.68)	0.56 (0.44, 0.66)	0.49 (0.39, 0.60)	0.47 (0.36, 0.58)
Q2 (5.45–10.4)	0.56 (0.45, 0.66)	0.57 (0.46, 0.67)	0.47 (0.37, 0.58)	0.48 (0.37, 0.59)	0.42 (0.32, 0.53)	0.42 (0.32, 0.53)
Q3 (10.5–24.7)	0.54 (0.43, 0.64)	0.54 (0.43, 0.64)	0.47 (0.37, 0.58)	0.47 (0.36, 0.58)	0.37 (0.27, 0.48)	0.36 (0.26, 0.47)
Q4 (24.8–306.0)	0.48 (0.38, 0.58)	0.50 (0.39, 0.60)	0.39 (0.29, 0.49)*	0.40 (0.30, 0.51)*	0.32 (0.23, 0.42)*	0.32 (0.22, 0.43)
*p*-Trend	0.14	0.26	0.03	0.07	0.03	0.07
MECPP (μg/L)
Q1 (≤ 14.5)	0.61 (0.50, 0.70)	0.59 (0.48, 0.69)	0.55 (0.45, 0.66)	0.54 (0.43, 0.64)	0.46 (0.36, 0.57)	0.43 (0.33, 0.54)
Q2 (14.6–26.2)	0.55 (0.44, 0.65)	0.55 (0.44, 0.66)	0.47 (0.37, 0.58)	0.48 (0.37, 0.58)	0.44 (0.34, 0.55)	0.43 (0.33, 0.54)
Q3 (26.3–56.6)	0.51 (0.41, 0.62)	0.52 (0.41, 0.62)	0.47 (0.37, 0.58)	0.47 (0.37, 0.58)	0.38 (0.28, 0.49)	0.37 (0.27, 0.48)
Q4 (56.7–760.5)	0.50 (0.40, 0.61)	0.51 (0.41, 0.62)	0.41 (0.31, 0.51)	0.42 (0.31, 0.52)	0.33 (0.24, 0.43)	0.32 (0.23, 0.43)
*p*-Trend	0.25	0.40	0.09	0.17	0.06	0.12
MEP (μg/L)
Q1 (≤ 21.4)	0.62 (0.52, 0.72)	0.60 (0.49, 0.70)	0.58 (0.48, 0.68)	0.55 (0.44, 0.66)	0.44 (0.34, 0.55)	0.40 (0.30, 0.51)
Q2 (21.5–49.2)	0.52 (0.42, 0.63)	0.53 (0.43, 0.64)	0.45 (0.35, 0.56)	0.46 (0.35, 0.56)	0.38 (0.29, 0.49)	0.38 (0.28, 0.49)
Q3 (49.3–128.1)	0.51 (0.41, 0.61)	0.52 (0.41, 0.62)	0.44 (0.34, 0.54)	0.44 (0.34, 0.55)	0.37 (0.27, 0.47)	0.36 (0.26, 0.47)
Q4 (128.2–3,481)	0.50 (0.40, 0.60)	0.52 (0.42, 0.63)	0.43 (0.33, 0.53)*	0.45 (0.34, 0.55)	0.40 (0.30, 0.51)	0.42 (0.31, 0.53)
*p*-Trend	0.25	0.55	0.17	0.40	0.87	0.65
MBP (μg/L)
Q1 (≤ 7.30)	0.63 (0.52, 0.72)	0.61 (0.50, 0.71)	0.55 (0.45, 0.65)	0.53 (0.42, 0.63)	0.48 (0.37, 0.58)	0.44 (0.34, 0.55)
Q2 (7.31–12.8)	0.57 (0.46, 0.66)	0.57 (0.46, 0.67)	0.50 (0.40, 0.60)	0.50 (0.40, 0.61)	0.41 (0.31, 0.52)	0.41 (0.31, 0.51)
Q3 (12.9–20.8)	0.47 (0.37, 0.57)*	0.48 (0.38, 0.59)	0.40 (0.30, 0.50)*	0.41 (0.31, 0.51)	0.36 (0.27, 0.47)	0.36 (0.27, 0.47)
Q4 (20.9–435.0)	0.50 (0.40, 0.60)	0.51 (0.41, 0.62)	0.45 (0.35, 0.55)	0.46 (0.36, 0.57)	0.35 (0.25, 0.45)	0.35 (0.25, 0.46)
*p*-Trend	0.10	0.22	0.17	0.36	0.09	0.19
MiBP (μg/L)
Q1 (≤ 3.50)	0.63 (0.52, 0.72)	0.62 (0.51, 0.72)	0.54 (0.44, 0.64)	0.53 (0.42, 0.63)	0.47 (0.36, 0.57)	0.44 (0.34, 0.55)
Q2 (3.51–6.63)	0.53 (0.43, 0.63)	0.53 (0.42, 0.63)	0.49 (0.39, 0.60)	0.48 (0.38, 0.59)	0.40 (0.30, 0.51)	0.39 (0.29, 0.49)
Q3 (6.64–12.0)	0.49 (0.39, 0.59)	0.51 (0.40, 0.62)	0.43 (0.33, 0.53)	0.44 (0.34, 0.55)	0.36 (0.27, 0.47)	0.37 (0.27, 0.48)
Q4 (12.1–49.7)	0.51 (0.41, 0.62)	0.52 (0.41, 0.63)	0.44 (0.34, 0.55)	0.44 (0.34, 0.55)	0.37 (0.27, 0.48)	0.36 (0.26, 0.47)
*p*-Trend	0.20	0.30	0.18	0.27	0.23	0.30
MBzP (μg/L)
Q1 (≤ 1.74)	0.55 (0.45, 0.65)	0.54 (0.43, 0.65)	0.50 (0.39, 0.60)	0.48 (0.38, 0.59)	0.41 (0.32, 0.52)	0.39 (0.29, 0.50)
Q2 (1.75–3.43)	0.58 (0.47, 0.68)	0.58 (0.47, 0.68)	0.48 (0.38, 0.59)	0.47 (0.37, 0.58)	0.37 (0.28, 0.48)	0.35 (0.25, 0.46)
Q3 (3.44–7.334)	0.54 (0.43, 0.64)	0.55 (0.45, 0.66)	0.48 (0.38, 0.59)	0.50 (0.39, 0.61)	0.46 (0.35, 0.56)	0.47 (0.37, 0.58)
Q4 (7.35–120.9)	0.50 (0.40, 0.61)	0.50 (0.40, 0.61)	0.44 (0.34, 0.55)	0.44 (0.33, 0.55)	0.36 (0.26, 0.47)	0.34 (0.25, 0.45)
*p*-Trend	0.37	0.44	0.44	0.51	0.53	0.59
MCPP (μg/L)
Q1 (≤ 1.80)	0.60 (0.50, 0.70)	0.60 (0.49, 0.70)	0.53 (0.43, 0.63)	0.52 (0.41, 0.63)	0.45 (0.34, 0.55)	0.43 (0.33, 0.54)
Q2 (1.81–3.19)	0.50 (0.40, 0.61)	0.50 (0.40, 0.61)	0.43 (0.33, 0.53)	0.42 (0.32, 0.53)	0.40 (0.30, 0.51)	0.39 (0.29, 0.50)
Q3 (3.20–6.58)	0.53 (0.42, 0.63)	0.53 (0.42, 0.63)	0.47 (0.37, 0.58)	0.47 (0.36, 0.57)	0.42 (0.32, 0.53)	0.41 (0.31, 0.52)
Q4 (6.59–202.2)	0.53 (0.43, 0.63)	0.55 (0.44, 0.65)	0.47 (0.37, 0.58)	0.48 (0.38, 0.59)	0.33 (0.24, 0.43)	0.32 (0.23, 0.43)
*p*-Trend	0.67	0.87	0.79	0.98	0.11	0.16
MCOP (μg/L)^*b*^
Q1 (≤ 8.93)	0.54 (0.43, 0.64)	0.54 (0.43, 0.65)	0.49 (0.38, 0.60)	0.50 (0.39, 0.61)	0.46 (0.35, 0.57)	0.47 (0.36, 0.58)
Q2 (8.94–22.2)	0.55 (0.44, 0.65)	0.54 (0.43, 0.65)	0.48 (0.37, 0.59)	0.47 (0.36, 0.58)	0.40 (0.30, 0.51)	0.37 (0.27, 0.49)
Q3 (22.3–57.7)	0.58 (0.47, 0.68)	0.57 (0.46, 0.68)	0.48 (0.38, 0.59)	0.47 (0.36, 0.58)	0.40 (0.29, 0.51)	0.38 (0.27, 0.49)
Q4 (57.8–1350.0)	0.52 (0.42, 0.63)	0.53 (0.42, 0.64)	0.47 (0.36, 0.57)	0.47 (0.36, 0.58)	0.35 (0.25, 0.47)	0.34 (0.24, 0.46)
*p*-Trend	0.79	0.85	0.78	0.83	0.23	0.25
MCNP (μg/L)^*b*^
Q1 (≤ 2.51)	0.52 (0.42, 0.63)	0.52 (0.41, 0.63)	0.44 (0.34, 0.55)	0.43 (0.33, 0.55)	0.42 (0.31, 0.53)	0.41 (0.30, 0.52)
Q2 (2.52–4.52)	0.56 (0.45, 0.66)	0.56 (0.45, 0.67)	0.50 (0.39, 0.61)	0.50 (0.39, 0.61)	0.41 (0.31, 0.53)	0.40 (0.29, 0.51)
Q3 (4.53–7.97)	0.58 (0.47, 0.68)	0.59 (0.47, 0.69)	0.51 (0.40, 0.61)	0.51 (0.40, 0.62)	0.40 (0.29, 0.51)	0.39 (0.28, 0.50)
Q4 (7.98–281.3)	0.52 (0.42, 0.63)	0.52 (0.41, 0.63)	0.47 (0.36, 0.57)	0.46 (0.35, 0.57)	0.39 (0.28, 0.50)	0.37 (0.27, 0.49)
*p*-Trend	0.83	0.81	0.98	0.98	0.68	0.70
Adjusted models control for maternal age (continuous), body mass index (continuous), smoking status (never, ever), and primary SART infertility diagnosis at study entry (female, male, unexplained). ^***a***^Tests for linear trend were performed using the median level of urinary phthalate metabolite in each quartile as a continuous variable in the model. ^***b***^38 missing values for MCOP and MCNP. *p-Value for comparison for a specific quartile against Q1 < 0.05.						

It is well appreciated that during the course of the study, urinary concentrations of some of the phthalate metabolites have decreased in the general population (Zota et al. 2014), and there may also be a temporal trend in IVF success. In our data set, urinary DEHP metabolite concentrations tended to be higher in earlier years, but calendar year was not significantly associated with IVF outcome. When we explored adjusting for calendar year in the multivariable models, the effect estimates remained similar and the conclusions remained the same (data not shown). To keep the most parsimonious model possible, we opted to exclude year in the final multivariable model.

## Discussion

In the present study we used the model of IVF to investigate human reproduction and pregnancy outcomes, ranging chronologically from the number and maturity of oocytes at retrieval, number of fertilized embryos, and embryo implantation to live birth. Urinary metabolites of DEHP and DiDP were associated with decreased oocyte yield and number of MII oocytes at retrieval, whereas only metabolites of DiNP and DiDP were associated with reduced fertilization rate. There is evidence that a decreased oocyte yield predicts poorer IVF outcomes (Ji et al. 2013; Sunkara et al. 2011). Urinary concentrations of DEHP metabolites were associated with reduced probability of clinical pregnancy and live birth. The magnitude of this reduction was clinically relevant. The adjusted proportion of live births in the highest quartile of ΣDEHP was 0.28 compared with 0.47 in the lowest quartile. Although urinary concentrations of other phthalate metabolites (i.e., MEP, MBP, MIBP, MBzP, MCPP, and MCNP) were not significantly associated with decreased probability of implantation, clinical pregnancy, or live birth, nearly all of the models showed small to moderate reduced likelihood of these outcomes when the highest quartile was compared with the lowest quartile.

There are few epidemiologic studies on associations of phthalates with rates of pregnancy and miscarriage (Heudorf et al. 2007). A recent publication on time to pregnancy in a U.S. prospective cohort (LIFE study) did not find associations between maternal urinary concentrations of 14 phthalate metabolites and fecundity (Buck Louis et al. 2014). They enrolled 501 couples from 2005 through 2009 who discontinued contraception and attempted to become pregnant. They used time to pregnancy to assess fecundity and to determine the number of menstrual cycles required for pregnancy confirmed by hCG. Possible explanations for differences in results between our study, which found associations between some urinary phthalate metabolites and reduced probability of pregnancy and live birth, and the LIFE study, which did not find associations of phthalate metabolites with reduced fecundity, include the choice of study population, differences in co-exposures, and the potential for unmeasured confounding. Our study included couples from a fertility clinic, whereas the LIFE study enrolled couples discontinuing contraception and attempting to become pregnant, excluding any couples with physician diagnosed infertility/sterility. The differences in results across studies may suggest that couples from an infertility clinic may represent a sensitive subpopulation to environmental chemicals, specifically phthalates. Other explanations for differences in results may be related to analytical methods used to measure phthalate metabolites which varied across the two studies and potential co-exposures to other chemicals in the two study populations.

Another recent publication including 430 couples enrolled in a Danish prospective cohort study (from 1992 through 1994) reported an association between urinary concentrations of phthalate metabolites and pregnancy loss (Toft et al. 2012). The authors explored associations of urinary phthalate metabolite concentrations with early pregnancy loss end points comparable with those measured in our study. In the Danish study, 128 women were enrolled after discontinuation of birth control and followed prospectively until a clinical pregnancy or for six menstrual cycles if there was no clinical pregnancy. Subclinical embryonic loss (referred to as early pregnancy loss) was determined through measurements of hCG in urine samples collected on the first 10 days of each menstrual cycle. By phone interview with the women, data were also collected on self-reported clinical spontaneous abortion. Pregnancy loss was increased among women in the upper tertile of urinary MEHP concentrations [adjusted odds ratio (OR) = 2.87; 95% CI: 1.09, 7.57] compared with the lowest tertile. When early and late pregnancy loss were analyzed in separate models, the OR for urinary MEHP concentrations and early pregnancy loss was 40.67 (95% CI: 4.48, 369.5) for tertile 3 compared with tertile 1, whereas late pregnancy loss was negatively associated with urinary MEHP (OR = 0.25; 95% CI: 0.05, 1.8 for the third tertile compared with the first tertile). For the other phthalate metabolites measured (MEP, MBP, MBzP, and oxidative metabolites of DEHP, MEOHP, and MEHHP) there were also elevated, nonsignificant, adjusted odds of early pregnancy loss [ranging from 1.13 (95% CI: 0.36, 3.59) for MEP to 1.64 (95% CI: 0.52, 5.20) for MBP and 3.11 (95% CI: 0.87, 11.09) for MBzP]. There were no statistically significant associations between the other measured urinary phthalate metabolites and odds for spontaneous abortion.

Although the results of the Danish study (Toft et al. 2012) and our study for DEHP metabolites are consistent, it should be cautioned that the early pregnancy loss end point in Danish women conceiving naturally was not the same as our end points of implantation, clinical pregnancy, and live birth. The authors of the Danish study urge caution when interpreting their results for MEHP because the incidence of early pregnancy loss in the first tertile was only 3%; thus they note that the very high OR for early pregnancy loss may be partially attributable to chance. Although both studies measured urinary phthalate metabolites in the concurrent conception cycle (measured on days 3 and 9 in our study, and day 10 in the Danish study), there were differences in analytical methods for the measurement of phthalate metabolites that may contribute to differences in results across studies.

Although implantation, clinical pregnancy, and live birth measured in women are not strictly comparable with pregnancy loss end points measured in experimental animals, studies in rats—albeit at doses much higher than those experienced by our study population (Heudorf et al. 2007)—support associations between exposure to phthalates and pregnancy loss. Further support for the biological plausibility of the associations between early pregnancy loss in the epidemiologic studies were studies in rats that showed that exposure to DEHP led to reductions in aromatase mRNA and protein levels leading to a decreased conversion of testosterone to estradiol (Lovekamp-Swan and Davis 2003) and a decrease in progesterone and estradiol in prepubertal female rats dosed with DEHP (Svechnikova et al. 2007). Human studies have shown that low pregnancy levels of estradiol and progesterone are associated with fetal loss (Schindler 2004), and thus DEHP may be operating through this hormonal pathway to increase the risk of early pregnancy loss. Our results raise concerns primarily about the potential of DEHP to result in lower probability of clinical pregnancy and live birth following ART.

One potential limitation of the present study is the generalizability of our findings to couples who conceive without medical assistance. However, couples with infertility represent 10–15% of U.S. couples, and the number of couples undergoing infertility treatment is increasing. The number of ART births in the United States more than tripled from 1996 to 2009 (ACOG Committee 1998; CDC/ASRM/SART 2012). Based on this trend, the number of U.S. children born through ART over the next 10 years is expected to be well over 1 million (CDC/ASRM/SART 2012). We also have indirect evidence to support the generalizability of these results based on previous work among this cohort of couples undergoing IVF that have either been consistent with, or since been corroborated by, other studies of non-IVF populations. Some examples include associations between phthalates and lower male steroid hormones (Meeker et al. 2009; Mendiola et al. 2011; Pan et al. 2006); pesticide exposure and semen quality (Meeker et al. 2008; Perry et al. 2007), thyroid hormone levels (Lacasaña et al. 2010; Meeker et al. 2006), and sperm DNA damage (Meeker et al. 2004; Xia et al. 2005); and self-reported maternal exposure to secondhand tobacco smoke and early pregnancy loss (Meeker et al. 2007a, 2007b; Peppone et al. 2009). Of interest, our decision to study the fertility clinic population was based on using an efficient design with sufficient power to investigate environmental influences on clinically relevant, yet previously unobservable, outcomes (e.g., fertilization rate and implantation) in a potentially vulnerable subpopulation. Thus, in summary, we believe that any potential limitations in generalizability in the proposed study are outweighed by its strengths because other study designs do not allow for the depth and breadth of exploration into these reproductive health effects in such a cost-efficient manner. In addition, other strengths include state-of-the-art exposure biomarker measures at the CDC, clinical outcomes from electronic medical records, and collection of data on potential confounders.

Another potential limitation of the study is misclassification of exposure based on urinary concentrations of phthalate metabolites. Single measures of urinary biomarkers of exposure to these short-lived compounds may not represent exposure during critical windows that may span weeks (i.e., implantation) or months (i.e., miscarriage, live birth). We have conducted a study (Braun et al. 2012), as have others (Hoppin et al. 2002; Peck et al. 2010; Preau et al. 2010), that demonstrates low to moderate reproducibility (intraclass correlations ranged from 0.15 to 0.65) of urinary levels of phthalate metabolites over time frames of weeks to a few months. Although it is well recognized that environmental exposures in close temporal proximity to the outcome are considered highly relevant, it is also important to consider that exposures distant from the outcome may also be relevant. For example, exposure very early in pregnancy may affect miscarriage later in pregnancy through effects on embryo quality and placentation. To reduce exposure misclassification in the present study, we collected two urine samples from each woman during each IVF cycle and used the geometric mean of the two cycle-specific urine concentrations as the exposure metric for the periconception exposure window.

In conclusion, using IVF as a model to investigate human reproduction and pregnancy outcomes, we found that concentrations of urinary metabolites of DEHP and DiDP were inversely associated with oocyte yield and number of MII oocytes at retrieval, whereas only metabolites of DiNP and DiDP were associated with reduced fertilization rate. Urinary concentrations of DEHP metabolites were negatively associated with likelihood of clinical pregnancy and live birth following IVF. These results highlight the potential reproductive effects of low-level exposure (i.e., background exposure levels of the general population) to phthalates and adverse IVF outcomes.
